# Effects of Repetitive Shoulder Activity on the Subacromial Space in Manual Wheelchair Users

**DOI:** 10.1155/2014/583951

**Published:** 2014-07-20

**Authors:** Yen-Sheng Lin, Michael Boninger, Lynn Worobey, Shawn Farrokhi, Alicia Koontz

**Affiliations:** ^1^Human Engineering Research Laboratories, VA Pittsburgh Healthcare System, University of Pittsburgh, Pittsburgh, PA 15206, USA; ^2^Department of Rehabilitation Science and Technology, University of Pittsburgh, Pittsburgh, PA 15260, USA; ^3^Department of Bioengineering, University of Pittsburgh, Pittsburgh, PA 15261, USA; ^4^Department of Physical Medicine and Rehabilitation, University of Pittsburgh, Pittsburgh, PA 15213, USA; ^5^Department of Physical Therapy, University of Pittsburgh, Pittsburgh, PA 15260, USA

## Abstract

This study investigated (1) the effect of repetitive weight-relief raises (WR) and shoulder external rotation (ER) on the acromiohumeral distance (AHD) among manual wheelchair users (MWUs) and (2) the relationship between shoulder pain, subject characteristics, and AHD changes. Twenty-three MWUs underwent ultrasound imaging of the nondominant shoulder in an unloaded baseline position and while holding a WR position before and after the WR/ER tasks. Paired *t*-tests and Spearman correlational analysis were used to assess differences in the AHD before and after each task and the relationships between pain, subject characteristics, and the AHD measures. A significant reduction in the subacromial space (*P* < 0.01) occurred when subjects performed a WR position compared to baseline. Individuals with increased years of disability had greater AHD percentage narrowing after WR (*P* = 0.008). Increased shoulder pain was associated with AHD percentage narrowing after ER (*P* ≤ 0.007). The results support clinical practice guidelines that recommend MWUs limit WR to preserve shoulder function. The isolated repetitive shoulder activity did not contribute to the changes of subacromial space in MWUs. The ultrasonographic measurement of the AHD may be a target for identifying future interventions that prevent pain.

## 1. Introduction

Subacromial impingement syndrome (SIS) is a common shoulder dysfunction in manual wheelchair users (MWUs). The mechanisms of SIS can be divided into intrinsic and extrinsic factors. [1–3] The intrinsic factors include rotator cuff degeneration, aging, arthritis, acromial shape, and abnormalities including subacromial and acromioclavicular joint spurs. Extrinsic factors include misalignment of the shoulder joint caused by muscle weakness or improper trunk postures, altered scapular kinematics, and mechanical compression from forces that drive the humeral head further into the glenohumeral joint, causing impingement of the supraspinatus tendon under the acromioclavicular arch and inflammation. Intrinsic and extrinsic factors may not be mutually exclusive and are exacerbated by overuse syndromes [2].

MWUs commonly experience overuse because their upper extremities are used extensively for mobility and activities of daily living (ADL). The weight-relief raise (WR) is an ADL that requires heavy and frequent shoulder loading. During a WR, MWUs need to lift and support the weight of the body to reduce pressure on the buttocks. This activity results in excessive shoulder joint loading and requires rotator cuff muscles to maintain glenohumeral joint stability [4–6]. van Drongelen et al. simulated shoulder joint reaction forces during the WR using musculoskeletal modeling techniques. They found that large weight-bearing forces (1288 N) acted to drive the humerus into the glenohumeral joint during the WR [6]. Gagnon et al. compared shoulder mechanical loads during WR and sitting pivot transfers among 13 MWUs with spinal cord injury (SCI). They reported that the bodyweight-normalized superior shoulder joint force during WR (2.91 N/kg) largely exceeded the amplitudes found during sitting pivot transfers in the leading arm (1.63 N/kg) and trailing arm (1.47 N/kg). Due to the limited size of the subacromial space, WR positioning is most likely to impinge the subacromial structures [7]. There is limited information on the impact of holding the WR position and isolated repetitive WR maneuvers on the subacromial space.

Shoulder external rotation (ER) is a commonly prescribed training among MWUs to strengthen the shoulder external rotators to act against potentially injurious forces during wheelchair activities [8]. Shoulder external rotators, including infraspinatus, supraspinatus, posterior deltoid, and teres minor, are important for maintaining glenohumeral joint positioning [9]. Previous studies have found MWUs with paraplegia have comparative weakness of shoulder external rotators compared to shoulder internal rotators, resulting in shoulder muscle imbalances [10]. Shoulder muscle imbalances can lead to functional instability of the glenohumeral joint, resulting in the subacromial space narrowing and placing the individual at a higher risk of developing SIS [11]. Previous studies have implied the narrowing of the subacromial space after isolated repetitive ER in subjects with SIS or rotator cuff tear. However, there is a knowledge gap regarding how the isolated repetitive ER contributes to subacromial space narrowing in the MWU population.

We recently described a reliable method to quantify the subacromial space by using ultrasound while holding a WR position [12]. Ultrasound has the advantage of enabling the shoulder to be scanned in a functional posture. The primary purpose of this study was to investigate the subacromial space with the shoulder in an unloaded neutral position (e.g., baseline) and in a WR position both before and within one minute after isolated repetitive WR and ER tasks. We hypothesized that the acromiohumeral distance (AHD), linear measurement of the subacromial space, in the WR position, would be narrower than the baseline AHD. We also hypothesized that the AHD would be narrower after subjects completed each protocol compared to before the protocol. A secondary goal of this study was to examine the relationship between shoulder pain, subject characteristics, and AHD.

## 2. Methods

### 2.1. Subjects

Study participants were a convenience sample recruited during the 2011 National Veterans Wheelchair Games (NVWG). For a power of 0.95, an *α* level of 0.05, and an effect size of 1.09 using a paired *t*-test, a sample size of 23 was required for this study. The effect size was calculated based on ultrasound data collected from wheelchair users in previous reliability study [12]. Inclusion criteria included using a manual wheelchair as primary means of mobility, able to perform at least 10 WR in a row without assistance, and between 18 and 65 years of age. The exclusion criteria included history of fractures or dislocations in the shoulder from which the subject had not fully recovered, upper limb dysesthetic pain as a result of a syrinx or complex regional pain syndrome, and history of cardiovascular or cardiopulmonary disease. Informed consent was obtained from all the subjects before participation in this study. The research protocol was approved by the Department of Veteran Affairs Institutional Review Board.

### 2.2. Questionnaires

Basic demographic information including age, height, weight, and date of injury/diagnosis was collected using self-report. All subjects completed the Wheelchair Users Shoulder Pain Index (WUSPI) [13]. The WUSPI is a reliable and validated 15-item self-report instrument that measures shoulder pain intensity in wheelchair users in the last seven days during various functional activities of daily living including transfers, wheelchair mobility, dressing, overhead lifting, and sleeping [14]. Each item is scored using a 10 cm visual analog scale anchored at the ends with the descriptors of “no pain” and “worst pain ever experienced.” Total score was calculated by summing the individual scores divided by the number of performed activities and then multiplying by 15 [15].

The OMNI pain scale is a numerical rating scale ranging from 0 to 10 [16–18]. The OMNI pain scale has been previously validated for walking, running, and cycle ergometer exercise and for use by male and female adults during upper and lower body resistance exercise. The OMNI scale was administered prior to the beginning of testing, to establish a baseline measure of pain, and after each activity, to determine the intensity of activity-induced pain experienced during the testing.

### 2.3. Procedure

Shoulder circumference and upper arm length were obtained from all subjects at the beginning of testing. The shoulder circumference and upper arm length were measured while the subjects were in the seated anatomical position. The shoulder circumference was measured from the superior portion of the acromion to the axilla. The upper arm length was measured from the most lateral and superior portion of the acromion to the tip of the olecranon. A single investigator conducted all of the measurements using a standard tape measure. Using this method to record similar anthropometrical measures has been found to be reliable [19].

Subjects transferred to a Biodex System 3 dynamometer (Biodex Medical System, Inc., Shirley, NY) with custom-made adjustable height armrests. Armrests were fitted to each subject to allow pushing straight up with full elbow extension to off load the buttock tissue. The seat height was fixed during the entire testing. The WR entailed lifting and holding the buttocks off the seat with an elbow locked position [4, 5]. The WR task was repeated at a rate of 30 repetitions per minute to the auditory cue of a metronome. Subjects were instructed to stop when they were no longer able to continue or until they completed two minutes of activity. The total number of WR raises (60) is similar to the number that would be performed each day in case of following the recommended frequency of pressure relief (one time every 15 to 30 minutes) [20].

The ER task followed a similar protocol to a previous study involving neurologically intact individuals without shoulder disorders and was designed to overuse the shoulder external rotators [21]. The nondominant side upper arm was adducted at the side with the elbow bent 90°. The subject was instructed to externally rotate the forearm from a shoulder neutral position to 45° or the maximum range of ER that they could reach comfortably [22]. The trunk was secured to minimize compensatory movements using straps from the Biodex that crossed the chest and lap. The strap has been used in previous studies to support targeted joint movements among spinal cord injured and able-bodied subjects [23, 24]. The dynamometer was adjusted to match the level of their tested and nontested shoulders before the ER activity. Resistance for the ER task was set for 5% of self-reported body weight [25]. To minimize the involvement of the shoulder internal rotators, the minimum resistance setting of 0.5 kg was used for the internal rotation direction from an externally rotated position back to neutral [26]. ER protocols were administered at the same pace and ended in the same manner as the WR task. The subjects rested in between the two protocols for a period of approximately 15 minutes.

### 2.4. AHD Ultrasound Examination

The subacromial space was quantified by measuring the AHD using ultrasound techniques as described in a previous reliability study [12]. The intrarater reliability of the AHD measurement with the shoulder in a neutral and WR position resulted in a standard error of measurement of 0.21 and 0.52 mm and intraclass correlation coefficient of 0.93 and 0.98, respectively [12]. A single examiner conducted all scans for each subject using a Philips HD11 1.0.6 ultrasound machine with a 5–12 MHz linear transducer. A water-based gel was applied on the skin to enhance conduction between the ultrasound probe and skin surface.

**Figure 1:**
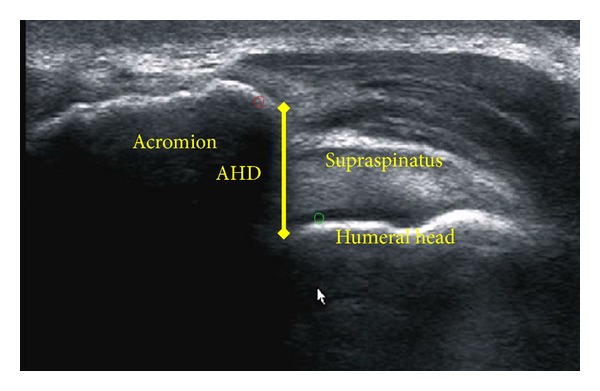
Ultrasonographic image of the acromiohumeral distance (AHD).

The nondominant side was chosen for all the AHD measures in order to minimize the effects caused by performing other types of activities of daily living on the dominant shoulder. The muscular demand of the nondominant shoulders among manual wheelchair users was also examined in previous studies [27]. The nondominant shoulder was scanned from the anterior aspect of glenoid to the flat segment of posterior acromion to capture the bright reflection of the bony contour of the acromion and humeral head (Figure 1). Ultrasound video was recorded at 60 Hz and scanning took approximately 10 seconds. A baseline US video was recorded with the shoulder in a neutral and resting position. Before and within one minute after each protocol, imaging was completed while the subject isometrically held the WR position [12]. We chose to examine AHD while subjects held the WR position because it provides a measure of what the AHD looks like under realistic, functional loading conditions.

### 2.5. Data Analysis

An investigator who was blinded to the timing of the video (e.g., pre or post) used a custom developed Matlab program to manually review each frame of the video and mark the inferior edge of acromion and superior margin of the humeral head. (Figure 1) The distance between the bony landmarks was calculated for each frame of the video and the narrowest distance across all frames was used for statistical analyses. A Shapiro-Wilk test indicated that the data followed a normal distribution. Therefore two-tailed paired *t*-tests were used to assess the difference in AHD between the baseline (unloaded) and WR shoulder positions as well as before and after performing the WR and ER tasks for all subjects (neutral versus pre-WR, pre-WR versus post-WR, pre-ER versus post-ER, and pre-WR versus pre-ER). A Bonferroni correction for multiple comparisons was applied, with a resultant level of significance of *P* < 0.013. Pearson's or Spearman's correlation was used to investigate relationships between the continuous measures, including AHD, AHD percentage changes (1), WUSPI score, OMNI scale score, and demographic data (e.g., height, weight, shoulder circumference, arm length, age, and years since acquiring the disability or injury). The strength of correlation was defined as a good to excellent relationship (*r* is above 0.75), moderate to good relationship (*r* = 0.50 to 0.75), fair relationship (*r* = 0.25 to 0.50), and little or no relationship (*r* = 0.00 to 0.25) [28]. Subject demographic variables statistically associated with the AHD measures were controlled for when testing the relationships between the AHD and pain measures. An alpha level less than 0.05 was established for significant changes:

(1) AHD  percentage  change  (%) =post-AHD  measure−pre-AHD  measurepre-AHD  measure  ×100%.

## 3. Results

### 3.1. Subjects

Twenty-three MWUs (twenty-two men and one woman) participated in this study. Sixteen MWUs had a spinal cord injury (five cervical and eleven thoracic), one had a unilateral transfemoral amputation, three had bilateral transtibial amputations, and three had multiple sclerosis. Twenty-two participants were right hand dominant. Descriptive data are provided in Table 1.

**Figure 2:**
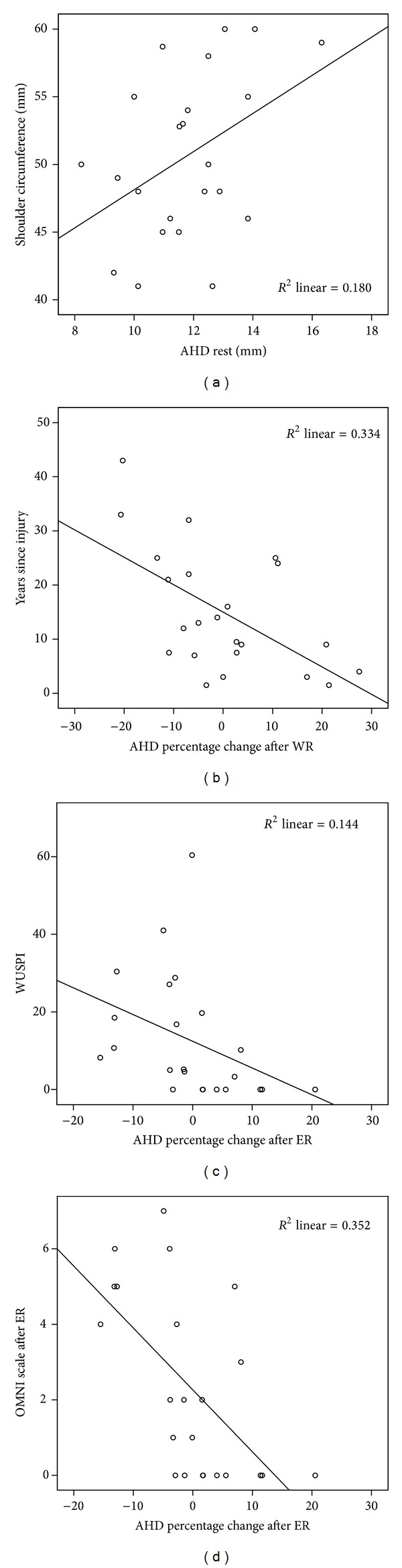
Correlation analysis for the AHD in neutral shoulder position with the shoulder circumference (*P* = 0.044, *n* = 23) (a), AHD percentage change after multiple weight-relief raises with years since injury (*P* = 0.008, *n* = 23) (b), AHD percentage change after shoulder external rotation activity with WUSPI (*P* = 0.007, *n* = 23) (c), and AHD percentage change with OMNI pain scale after ER (*P* = 0.003, *n* = 23) (d).

There were no significant differences in the AHD before and after performing WR (*P* = 0.89) and ER (*P* = 0.81) (Table 2). The AHD in the pre-WR and pre-ER positions were not different (*P* = 0.38) but both were significantly smaller than the AHD in the baseline shoulder neutral position (*P* < 0.001). No relationship between baseline AHD and age, height, weight, or arm length was found. Individuals with narrower AHD at baseline had smaller shoulder circumferences (*r* = 0.42, *P* = 0.044, Figure 2(a)). Individuals with increased years of disability had greater AHD percentage narrowing after the WR task (*r* = −0.54, *P* = 0.008, Figure 2(b)). More shoulder pain on WUSPI was associated with greater percentage narrowing of the AHD after the ER task (*r* = −0.41, *P* = 0.007, Figure 2(c)). The OMNI pain scale results measured at baseline, after WR, and after ER were 1.04 ± 1.58, 2.09 ± 2.56, and 2.30 ± 2.42, respectively. Individuals with higher scores on the OMNI pain scale after ER had greater percentage narrowing of the AHD after ER (*r* = −0.59, *P* = 0.003) (Figure 2(d)).

## 4. Discussion

The results of this study indicate that AHD narrowing occurs when MWUs assume a weight-bearing position with their arms. When our subjects assumed the WR position, a statistically significant reduction in space occurred. In this position, the elbows are in full extension allowing the humeral head to be oriented more directly upward and into the joint while the scapula is anteriorly tilted and internally rotated [29]. The humeral head migration, scapular anterior tilting, and internal rotation as well as the large superior or posterior weight-bearing forces likely contribute to a reduction in subacromial space [30]. This narrowing of the AHD can lead to commonly experienced pathologies among MWUs, including enlarged bursa, tendon inflammation, or irregularities of the gliding surface [31]. Weight-bearing positions are difficult to avoid and occur daily at high frequency during wheelchair transfers and pressure relief, causing wheelchair users to be at risk for developing SIS. These findings support clinical practice guidelines that recommend MWUs limit the WR technique for pressure relief [8].

In contrast to our hypothesis, this study did not find differences in the AHD measures before and after isolated repetitive WR. One possible explanation is that not all subjects may have experienced overuse in the targeted rotator cuff muscles. Many individuals in our study were not able to complete two minutes of activity which could have been a result of fatigue or other reasons (e.g., pain onset, discomfort, boredom, etc.). Also, the triceps are the most active muscle group during a WR task and overuse would most likely occur in this muscle first [4]. In addition, a fine-wire electromyography (EMG) study showed that the rotator cuff and depressor muscles (e.g., supraspinatus, infraspinatus, subscapularis, and serratus anterior) were minimally active during the WR task (less than 25% of maximum voluntary contraction) whereas moderate to high activity was found for the sternal pectoralis major and latissimus dorsi muscle groups [4]. Activation of these muscles is believed to help transfer humeral loads onto the trunk, functionally circumventing the glenohumeral joint, and reducing the potential for impingement. Thus, it may be more difficult to detect changes in the AHD when the shoulder is in the WR position.

The ER activity is an overuse protocol targeting the external rotators and minimizing involvement of other shoulder muscles. Previous studies have shown superior migration of the humeral head occurs after overusing the shoulder external rotators [32]. However, we found no difference in the AHD after the task among MWUs except in subjects who had greater levels of shoulder pain. This difference could be because prior studies on neurologically intact individuals measured subjects with their arms in a nonweight-bearing position during scapular plane abduction. With arm elevation, the deltoid muscle enhances the upward pull of the humerus. Increased humeral head migration has been reported when the inefficient force of the fatigued rotator cuff muscles could not counteract the superior force from the deltoid muscle [33]. Active shoulder elevation would likely magnify the upward shift of the humeral head after the isolated repetitive ER in contrast to the WR position. Further studies are needed to understand if there are other arm positions that would be more sensitive to detecting changes with the overuse of shoulder external rotators.

Not finding differences in AHD after the isolated repetitive tasks may also result from compensatory scapular motions [34] and motor strategies or muscle firing patterns [35] used to preserve the subacromial space [36]. A recent meta-analysis reported that subjects with SIS often demonstrate altered three-dimensional scapular behavior [37], including decreased scapular posterior tilt, upward rotation, and external rotations, which may negatively impact the subacromial space [38–40]. However, other investigations have reported different behavior, such as increased upward rotation scapular rotation, suggesting these motions are compensatory mechanisms to avoid further shoulder pain during activity [34]. Increased upward rotation and external rotation of the scapula, which would have a positive effect on the AHD, have also been linked to inadequate rotator cuff function [41]. It is possible that in response to the overuse our subjects were demonstrating compensatory scapular kinematic patterns in favor of protecting the subacromial space [4]. Future studies are warranted to understand the relationship between scapular motions during isolated repetitive shoulder tasks and changes in the subacromial space.

Our study found that more shoulder pain was related to greater AHD percentage narrowing after ER. Not finding the same association after the WR task may point to the effectiveness of the ER task in targeting overuse of the infraspinatus muscle [42]. As mentioned, SIS has been associated with scapular kinematics, which reduce the subacromial space. However how pain influences scapular kinematics is not well understood. A recent study anesthetized painful impinged shoulders and found greater anterior tipping of the scapula during greater humeral elevation angles, which can further reduce the subacromial space [43]. Thus pain may help protect the space from greater narrowing to some degree. However our study found that greater amounts of pain did not hinder narrowing of the space after repetitive ER activity in our sample of wheelchair users. The AHD measure may be useful in the future for evaluating the effectiveness of interventions that are targeted at reducing shoulder pain among wheelchair users.

Our results were consistent with other studies that found that AHD measures were not significantly correlated with the characteristics commonly linked to SIS such as age and weight [44, 45]. However a positive correlation was found between percentage narrowing of the AHD after WR with years of injury/diagnosis. Several studies have found that a longer duration of wheelchair use is associated with greater pain and shoulder pathology [8, 46, 47]. In addition to shoulder pathology, other problems commonly seen in long-term wheelchair users, including muscle strength imbalances around the shoulder, joint instability, altered scapular kinematics and abnormal glenohumeral motion, and subluxation, likely contributed to a greater reduction in the space following the WR task [48].

Our study had several limitations. Because our protocol was conducted at a national wheelchair sporting event, it was difficult to control for the amount of upper limb activity experienced before the testing. We conducted the informed consent process and questionnaires at the beginning of the study (e.g., a process that took 15–20 minutes) which helped to provide some washout period for the participants before starting the protocol. Also the within-subject design helped to control the effects that varying amounts of preactivity may have had on the primary pre-/post-AHD measures. In addition, wheelchair users who participate in sporting events may be considered more active than the general population. However, Tolerico et al. found that veterans who participate in the NVWG are not significantly different with respect to mobility characteristics and activity levels from their community-dwelling wheelchair using counterparts [49]. Another limitation is that the two activities were performed in order (WR followed by ER) on the same day and it is possible that there was not enough recovery time to compensate for the overuse on the muscles. It is reassuring that the two AHD measures taken before each task were not significantly different.

Finding significant relationships between AHD changes and pain in our study implies that there is clinical relevancy with the AHD measure. However, because we did not specifically target symptomatic subjects for this study or study the effects of an intervention, more work is needed to define clinically meaningful changes in the AHD for manual wheelchair users. Limited research has been done so far in a wheelchair user population. A previous study on nonwheelchairs found that the ultrasonographic measurement of the AHD in affected shoulders among individuals with SIS (19.4 mm) was significantly narrower than the AHD in their nonaffected shoulder (22.2 mm, *P* < 0.001) [45]. Another study found statistical significance for mean differences in the AHD that ranged from 1.7 to 2.1 mm before and after a scapular assistant test designed to change scapular position and subacromial space among symptomatic subjects. Both of these studies suggest that there may be clinical relevancy with ultrasound AHD changes on the order of 1.7 to 2.8 mm. This is within the realm of the statistically significant mean differences found in the AHD between the neutral and WR position. Other variables such as acromial shape, abnormal scapular kinematics, and impaired rotator cuff function were not investigated and could be additional sources to explain AHD narrowing. Acute changes were examined with the shoulders in a loaded position and differences may have been more apparent had the arm been scanned in an elevated, unloaded position. As scapular orientation has also been shown to affect AHD [50], future work should investigate scapular and humeral positioning to gain further insight into injury mechanisms.

## 5. Conclusions

The results of this study suggest that MWUs should limit WR for pressure relief, as placing the shoulder in a WR position led to a significant reduction in the subacromial space. The isolated repetitive shoulder activity did not contribute to the changes of subacromial space in MWUs. This study provides objective evidence that the AHD is associated with pain and long-term use of a wheelchair. Ultrasonographic measurement of the AHD may be useful for identifying interventions that prevent pain. A better understanding of the scapular and humeral kinematics may help to elucidate mechanisms leading to subacromial impingement in wheelchair users.

## Figures and Tables

**Table 1:** Subject demographics (*n* = 23).

Demographic	Mean ± standard deviation	Range
Age	46 ± 12	26–64
Height (m)	1.78 ± 0.08	1.65–1.93
Weight (kg)	81 ± 18	55–130
Time since injury (year)	15 ± 10	1.5–33.5
Number of WR	34 ± 16	10–61
Number of ER	39 ± 18	6–60
WUSPI	14.08 ± 18.07	0–60, median 12.6
OMNI pain scale baseline	1.04 ± 1.58	0–5
OMNI pain scale after WR	2.09 ± 2.56	0–8
OMNI pain scale after ER	2.30 ± 2.42	0–7

**Table 2:** AHD for each subject.

Disability	Rest	Multiple weight-relief raises (mm)		Shoulder external rotation activity (mm)	
		Pre	Post	Pre	Post
C3 spinal stenosis	14.07	10.85	10.85	10.67	9.31
T4 com. SCI	11.53	9.32	11.27	9.18	9.04
C6 inc. SCI	10.96	10.14	9.03	8.77	8.90
MS	12.64	8.08	9.45	11.25	10.82
Amp (LAK)	12.88	12.76	10.17	10.17	10.34
T4 com. SCI	11.51	10.00	9.31	9.72	10.82
Amp (RBK, LAK)	12.37	9.83	11.93	9.32	10.41
T7 inc. SCI	11.64	10.28	11.37	10.70	10.69
T9 inc. SCI	12.50	11.64	11.51	11.67	11.10
MS	9.32	9.04	11.53	10.27	8.92
C3 inc. SCI	10.96	10.55	10.83	10.00	8.45
MS	12.36	9.03	7.16	8.47	8.36
T12 com. SCI	9.44	10.00	9.31	10.00	9.73
T12 inc. SCI	11.81	10.00	10.27	10.27	10.69
Amp (RAK, LAK)	10.00	8.38	9.31	7.95	8.08
T12 com. SCI	10.14	9.31	8.77	8.75	8.49
Amp (RAK, LBK)	10.14	8.49	7.36	7.50	9.04
C5 inc. SCI	13.06	11.39	10.82	11.39	10.95
C7 inc. SCI	16.32	10.86	9.66	10.52	10.17
T10 inc. SCI	13.83	12.71	12.28	12.41	13.10
T11 inc. SCI	11.22	10.27	9.45	10.14	10.96
T12 com. SCI	13.84	11.10	11.51	9.73	10.41
T9 inc. SCI	8.22	6.03	6.08	6.71	5.83

Group mean	11.78 ± 1.83^†,‡^	10.00 ± 1.51^†^	9.97 ± 1.60	9.81 ± 1.36^‡^	9.77 ± 1.47

SCI, spinal cord injury (com., complete; inc. incomplete); Amp, amputee; RAK, right leg above knee; RBK, right leg below knee; LAK, left leg above knee; LBK, left leg below knee; MS, multiple sclerosis. ^†,‡^ *P* < 0.05.
